# Prostate-specific antigen (PSA) screening and follow-up investigations in Māori and non-Māori men in New Zealand

**DOI:** 10.1186/1471-2296-15-145

**Published:** 2014-08-26

**Authors:** Zuzana Obertová, Nina Scott, Charis Brown, Fraser Hodgson, Alistair Stewart, Michael Holmes, Ross Lawrenson

**Affiliations:** Waikato Clinical School, University of Auckland, Hamilton, New Zealand; Te Puna Oranga, Waikato District Health Board, Hamilton, New Zealand; School of Population Health, University of Auckland, Auckland, New Zealand; Urology Department, Waikato Hospital, Hamilton, New Zealand; Waikato Clinical School, Peter Rothwell Academic Centre, Private Bag 3200, Hamilton, 3240 New Zealand

**Keywords:** Prostate cancer, Ethnicity, Inequality, Biopsy, Specialist referral

## Abstract

**Background:**

Māori men in New Zealand have higher mortality from prostate cancer, despite having lower incidence rates. The objective of this study was to examine patterns of screening for prostate cancer in primary care and follow-up investigations after an elevated prostate-specific antigen (PSA) result in Māori and non-Māori men in order to help explain the observed differences in incidence and mortality.

**Methods:**

Men aged 40+ years were identified from 31 general practices across the Midland Cancer Network region. Computerised practice records were cross-referenced with laboratory data to determine the number and value of PSA tests undertaken between January 2007 and December 2010. Screening rates were calculated for the year 2010 by age, ethnicity, and practice. For men with an elevated PSA result information on specialist referrals and biopsy was extracted from practice records. Practice characteristics were assessed with respect to screening rates for Māori and non-Māori men.

**Results:**

The final study population included 34,960 men aged 40+ years; 14% were Māori. Māori men were less likely to be screened in 2010 compared with non-Māori men (Mantel Haenszel (M-H) age-adjusted risk ratio (RR), 0.52 [95% CI, 0.48, 0.56]). When screened, Māori men were more than twice as likely to have an elevated PSA result compared with non-Māori men (M-H age-adjusted RR, 2.16 [95% CI, 1.42, 3.31]). There were no significant differences between Māori and non-Māori men in the rate of follow-up investigations and cancer detection. Māori provider practices showed equal screening rates for Māori and non-Māori men, but they were also the practices with the lowest overall screening rates.

**Conclusions:**

Māori men were half as likely to be screened compared to non-Māori men. This probably explains the lower reported incidence of prostate cancer for Māori men. Practice characteristics had a major influence on screening rates. Large variation in screening behaviour among practices and differences in follow-up investigations for men with an elevated PSA result seems to reflect the uncertainty among GPs regarding PSA screening and management.

## Background

Prostate cancer has been identified as the primary contributor to cancer burden in New Zealand men; with prostate cancer burden for Māori men being 50% higher compared with non-Māori men [1]. Māori men were reported to have higher mortality from prostate cancer, despite having lower incidence rates [2–4].

Although screening for prostate cancer is not recommended in New Zealand due to the controversy about its harms versus benefits [5–7], it is common in New Zealand general practice [8, 9]. Screening rates in New Zealand are similar to other countries with privatised primary care services, such as the USA, Canada and Australia [10–12]. In 2010, approximately 80% of prostate-specific antigen (PSA) tests were undertaken in asymptomatic men [13], which means that by definition they were screening tests [14]. Despite the limitations of the PSA test when used as a screening tool, it is currently considered to be the best single test for early detection of prostate cancer [15].

A significant proportion of general practitioners (GPs) in New Zealand believes that screening for prostate cancer is beneficial [13, 16]. A recent study has found that 80% of PSA testing in New Zealand primary care was initiated by GPs, not by patients [13]. Similar screening behaviour and beliefs have been reported from other countries [17–19].

In the USA, differences in screening practices, such as availability and interval of screening contribute to ethnic disparities in prostate cancer incidence and mortality [20]. It is unclear whether the low prostate cancer incidence and high mortality for Māori men are due to disparities in screening or biopsy rates, resulting in later tumour stage at diagnosis for Māori men, and/or over-detection of indolent cancers for non-Māori men.

An in-depth understanding of the patterns of PSA screening in primary care is needed in order to explain the differences in prostate cancer incidence and mortality between Māori and non-Māori men in New Zealand so that evidence-based interventions can be developed to eliminate unfair and avoidable inequities.

The aim of this study was to ascertain annual PSA screening rates for Māori and non-Māori men using computerised records from general practices linked with laboratory data. Men with elevated PSA result were followed-up to examine management pathways, including referral and biopsy rates by ethnicity. The influence of practice characteristics on difference in screening rates between Māori and non-Māori men was also explored.

## Methods

### Study population

A cohort of men aged 40+ years enrolled in 31 general practices in the Midland Cancer Network (MCN) region was identified from computerised practice records. Each general practice provided baseline data (National Health Index code, date of birth, ethnicity) on all male patients aged 40+ years enrolled with the practice in 2010. Practices were purposefully selected to ensure sufficient numbers of Māori men and geographical coverage of the MCN region, which covers three District Health Boards (DHBs) of Waikato, Bay of Plenty and Lakes. There were approximately 150 general practices in the MCN region at the time of research.

### Data sources

All practices used the Healthtech Medtech software for recording patient data, clinical notes, and laboratory results. Men with a PSA test in 2010 were identified by cross-referencing data from the computerised practice records with laboratory data. Data linkage was based on the National Health Index (NHI) code, a unique identifier for people using health services in New Zealand.

All PSA results for the period from 1 January 2007 to 31 December 2010 were obtained from three community laboratories serving the MCN region; with more than 90% of tests being undertaken in one laboratory (Pathlab).

Data on prostate cancer registrations for the years 1994 to 2011 were available from the New Zealand Cancer Registry (NZCR). The NZCR collects data on all new cases of malignant cancers excluding squamous cell carcinoma and basal cell carcinoma of the skin. By linking the NHI numbers of the NZCR and the practice records, 1,006 men, of whom 69 were Māori (6.9%) with a prostate cancer diagnosis prior to 1 January 2010 or prior to any PSA test in 2010 were identified. These men were excluded from further analyses.

### Outcome variables

In order to calculate screening rates for prostate cancer, all men with a PSA test between 1 January 2010 and 31 December 2010 were identified. In men with more than one PSA test during 2010, the earliest test result was considered in the analysis. When one of the test results (not necessarily the earliest one) was elevated, the man was categorised as having an elevated PSA test during 2010. PSA values were classified as being elevated when they exceeded age-specific levels adapted from Oesterling et al. [21], as used by the local laboratories (40-49 years: >2.5 ng/ml; 50-59 years: >3.5 ng/ml; 60-69 years: >4.5 ng/ml; 70-79 years: >6.5 ng/ml; 80+ years: >7.0 ng/ml).

Men were classified as being screened when they had no elevated PSA result or prostate biopsy in the three years prior to 2010. In addition, men with elevated PSA result in 2010 were considered to be screened when there was no record of symptoms (urinary tract symptoms, erectile dysfunction, bone pain) for that year in the computerised practice records.

For men with an elevated PSA result, data on follow-up investigations, including specialist referral and biopsy were extracted. The NHI codes of men with elevated PSA result were also cross-referenced with pathology reports made available by the laboratories to ascertain data completeness regarding whether biopsy was undertaken, and what the result was. Men were followed up until 31 December 2011.

### Predictor variables

Ethnicity was extracted from general practice records. Patients who enrol with a general practice in New Zealand are asked to report their ethnicity along with other personal information. Men were categorised as either Māori or non-Māori. In the latter group, 91.9% were European, 2.4% Pacific, 3.3% Asian, and 2.4% of other ethnicity. Patients’ age was grouped into four categories (40-69, 60-69, 70-79 and 80+ years) for the assessment of screening rates. Age was collapsed into two categories (-40-59 years, 70+ years) for assessing the rates of follow-up investigations.

Individual practices were categorised by several parameters: Practices governed by Māori organisations were labelled Māori provider practices and were compared to other (not Māori provider) provider practices. Māori provider practices offer primary health care services rooted in Māori cultural practices, customs and world view.

Māori provider practices were then compared to other provider practices with respect to their size, including number of men aged 40+ years enrolled in the practice (<500 versus 500+), and number of GPs in the practice (1-3 versus 4+), and the proportion of Māori men aged 40+ years (<20% versus 20%+). In addition, practices were categorised by screening rate (<20% versus 20% + screened men aged 40+ years).

### Statistical analysis

Statistical analysis was performed with SPSS 19.0 and STATA IC/12 and included the Mantel-Haenszel (M-H) age-adjusted risk ratios (RR) calculated to compare screening rates, rates of elevated PSA results, follow-up investigations and cancer detection for Māori and non-Māori men. Mantel-Haenszel (M-H) age-adjusted risk ratios (RR) for Māori compared to non-Māori men were also calculated by practice.

Access to computerised records of general practices was obtained through close cooperation with general practitioners and practice managers. Ethical approval was issued by the Northern Y Ethics Committee (NTY/11/02/019). Māori consultation included the Midland Cancer Network Māori Advisory Group Hei Pa Harakeke, and Te Puna Oranga, the Waikato District Health Board Māori Unit.

## Results

### Screening rates and follow-up investigations

**Figure 1:**
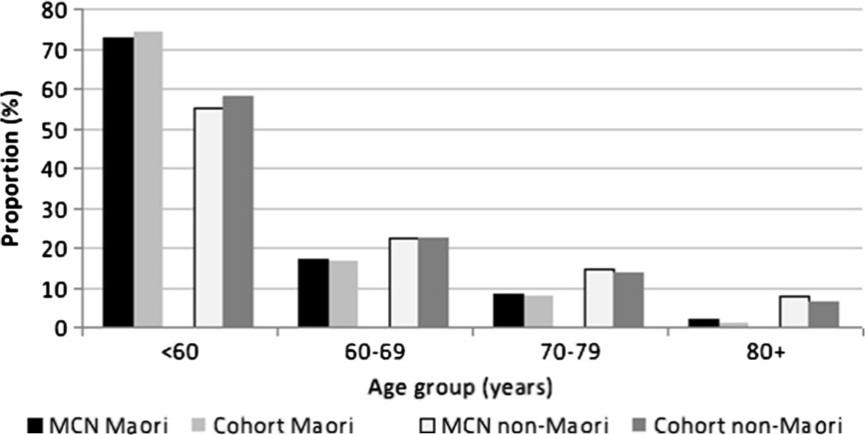
**Age distribution in Midland Cancer Network (MCN) region and our cohort by ethnicity.**

The preferential sampling of practices with high Māori population has proven successful as shown by comparing the demographic characteristics of this cohort with those of the MCN male population aged 40+ years. In 2010, there were 36,740 men enrolled in the 31 general practices, accounting for 25% of the estimated total male population aged 40+ years in the MCN region in 2010 [22]. Māori men comprised 14.3% (4986) of the final cohort. The estimated proportion of Māori men aged 40+ years residing in the MCN region in 2010 was 14.4% [22]. The age structure of Māori and non-Māori men of this cohort was similar to that of the total male population aged 40+ years in the MCN region (Figure 1).

The final study population included 34,960 men aged 40+ years after men diagnosed with prostate cancer before 2010 (1006) and men with unknown ethnicity (774) were excluded. Table 1 shows cohort characteristics of Māori and non-Māori men from 31 general practices.

**Table 1:** **Characteristics of the cohort of Māori and non-Māori men from 31 general practices**

	Māori		Non-Māori	
	(N = 4986)		(N = 29974)	
	n	%	n	%
**Age**				
40-59 years	3749	75.2	17874	59.6
60-69 years	805	16.1	6613	22.1
70-79 years	374	7.5	3814	12.7
80+ years	58	1.2	1673	5.6
**PSA test in 2010**	650	13.0	8066	26.9
**PSA test between 2007 and 2009** (% of tested men)	303	46.6	5092	63.1
**PSA level** (% of tested men)				
0-3.99 ng/ml	554	85.2	6824	84.6
4-9.99 ng/ml	71	10.9	976	12.1
10-19.99 ng/ml	18	2.8	189	2.3
20+ ng/ml	7	1.1	77	1.0

Māori men were younger than non-Māori men, which is consistent with the age structure differences between total Māori and non-Māori population in New Zealand [23]. Māori men were less likely to have a PSA test in 2010, and they were also less likely to have a PSA test in the three years prior to 2010 compared with non-Māori men.

The screening rate for Māori men was 11.2% compared with 22.6% for non-Māori men, thus Māori men were 48% less likely to be screened compared with non-Māori men (M-H age-adjusted RR, 0.52 [95% CI, 0.48, 0.56]; Table 2). When screened, 168 men had an elevated PSA result. There were 4.3% of Māori men and 2.1% of non-Māori men with an elevated PSA result (M-H age-adjusted RR, 2.16 [95% CI, 1.42, 3.31]; Table 2).

**Table 2:** **Screening rates in 2010 and proportion of men with elevated PSA result by age and ethnicity**

	Screening in 2010		Men with elevated PSA result ()	
	n (%)		n (% of screened men)	
	Māori	Non-Māori	Māori	Non-Māori
40-59 yrs	350	3385	12	52
	(9.3)	(18.9)	(3.4)	(1.5)
60-69 yrs	146	2095	7	64
	(18.1)	(31.7)	(4.8)	(3.1)
70-79 yrs	55	1055	5	21
	(14.7)	(27.7)	(9.1)	(2.0)
80 + yrs	6	251	0	7
	(10.3)	(15.0)	(0)	(2.8)
Total	557	6786	24	144
	(11.2)	(22.6)	(4.3)	(2.1)
Mantel-Haenszel age-adjusted RR	0.52 [0.48, 0.56]		2.16 [1.42, 3.31]	
[95% CI]				

In total, 66 (39.3%) men were referred after an elevated PSA result and 44 (66.7%) had a biopsy following referral. Of the men who underwent a biopsy, 27 (61.4%) were found to have cancer. Table 3 summarises age-specific referral, biopsy rates and results for Māori and non-Māori men. There were no significant differences between Māori and non-Māori men in the rates of follow-up investigations and cancer detection (Table 3). Cancer detection rate from men at risk (with elevated PSA result) was 167 per 1000 for Māori men and 160 per 1000 for non-Māori men. Cancer detection rate from all screened men was 7 per 1000 for Māori men and 3 per 1000 for non-Māori men.

**Table 3:** **Frequency of follow-up investigations and prostate cancer diagnosis in screened men with an elevated PSA result by age and ethnicity**

	Māori	Non-Māori
Referral (% of men with elevated PSA result)	n/N (%)	n/N (%)
40-69 years	9/19 (47.4%)	44/116 (37.9%)
70+ years	2/5 (40.0%)	11/28 (39.3%)
Total	11/24 (45.8%)	55/144 (38.2%)
Mantel-Haenszel adjusted RR [95% CI]	1.2 [0.74, 1.94]	
**Biopsy (% of referred men)**		
40-69 years	6/9 (66.7%)	33/44 (75.0%)
70+ years	1/2 (50.0%)	4/11 (36.4%)
Total	7/11 (63.6%)	37/55 (67.3%)
Mantel-Haenszel adjusted RR [95% CI]	0.94 [0.58, 1.50]	
**Positive biopsy (% of men with biopsy)**		
40-69 years	3/6 (50.0%)	19/33 (57.6%)
70+ years	1/1 (100%)	4/4 (100%)
Total	4/7 (57.1%)	23/37 (62.2%)
Mantel-Haenszel adjusted RR [95% CI]	0.90 [0.46, 1.73]	

### Practice characteristics and disparities in screening rates

Māori provider practices were predominantly smaller with respect to the number of enrolled men aged 40+ years, and to the number of general practitioners in the practice compared with other provider practices. In each of the Māori provider practices, Māori men represented 20 +% (ranging from 22% to 75%) of the enrolled men, while only one-fourth of the other providers had 20 +% of Māori men in their practice (the proportion ranged from 4% to 35%). Only one of the Māori provider practices (12.5%) was characterised as high-screening (with an overall screening rate of 20+ %) compared with 13 (59.6%) of the other provider practices.

**Figure 2:**
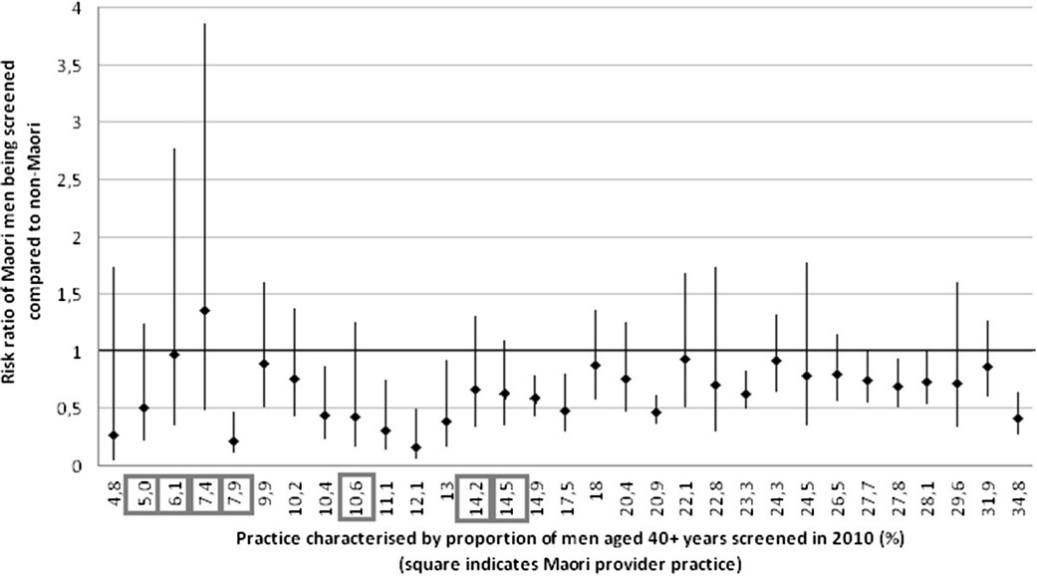
**Risk ratio of Māori men being screened by practice characterised by proportion of men aged 40+ years screened in 2010.**

The age-adjusted RR for Māori men being screened (compared with non-Māori men) by practice characterised by the overall proportion of screened men is depicted in Figure 2. There was a seven-fold variation in screening rates among practices (4.8% to 34.8%). There were two practices that had a RR of Māori and non-Māori screening rates close to (0.97) or above 1 (1.36), both of which were Māori provider practices (Figure 2).

In Māori provider practices Māori men were 34% less likely to be screened compared with non-Māori men (M-H age-adjusted RR, 0.66 [95% CI, 0.51, 0.85]), while in other practices Māori men were 45% less likely to be screened compared with non-Māori men (M-H age-adjusted RR, 0.55 [95% CI, 0.50, 0.60]).

## Discussion

Māori men were half as likely to be screened for prostate cancer compared with their non-Māori peers. Lower screening rates have been commonly associated with lower prostate cancer incidence rates [11, 20, 24, 25]. Several New Zealand studies have pointed out that incidence rates for prostate cancer are lower in Māori men compared with non-Māori men [26–28]. Our population-based study is the first to show that lower PSA screening rates are a significant factor contributing to lower prostate cancer incidence rates for Māori men.

Only 2% of screened men had an elevated PSA, so an overwhelming majority was shown to have a normal PSA result. However, screened Māori men were twice as likely to have an elevated PSA result compared with non-Māori men. This finding may be partly due to the fact that Māori men were less likely to have been tested in the three years prior to 2010, implying that there is a greater potential for detecting prostate issues in Māori men compared with more frequently tested non-Māori men.

There were no significant differences in the rates of follow-up investigations and cancer detection between Māori and non-Māori men with an elevated PSA result. In general, 39% of men with an elevated PSA result were referred to a specialist in the follow-up period, meaning that the majority of these men were managed in general practice. One of the reasons why general practitioners (GPs) do not immediately refer men with elevated PSA result may be their awareness of the fact that an increase in PSA level can be caused by prostatic diseases other than prostate cancer, such as prostatitis and benign prostatic hyperplasia [29, 30]. GPs may feel that repeated PSA tests are needed to assess the actual risk of prostate cancer before referring to a specialist.

Overall, 67% of referred men went on to have a biopsy. It is recommended that the decision when to proceed to prostate biopsy should be primarily based on PSA level and the result of digital rectal examination, but other factors, including patient’s age, family history, and co-morbidities may be considered by the specialist [6, 31]. These factors may also influence GPs’ decision to refer for a specialist assessment in the first place.

In our study, there was a large variation between practices in PSA screening rates. This finding is consistent with studies from other countries [32–34]. It has been shown that besides patient characteristics, such as age and co-morbidities, GP and practice characteristics, including the number of GPs in the practices and the number of patients seen by the GP influence screening behaviour [17, 35–37]. In addition, studies on screening behaviour of physicians report that GPs believe in the benefits of PSA screening, which is probably one of the reasons why they commonly initiate it [13, 17, 35]. It has also been shown that GPs expressed difficulties in understanding and explaining to patients the complexities of screening for prostate cancer [13, 17, 38]. Such difficulties have been attributed to the lack of consensus in screening guidelines between different national organisations as well as internationally [18, 39]. For example, at the time of our research the Urological Society of Australia and New Zealand [40] has recommended that asymptomatic men aged 55-69 years should be offered a prostate-specific antigen (PSA) test and digital rectal examination (DRE) after the patients have been advised on the risks and benefits of PSA screening, while in contrast, the Ministry of Health (The National Screening Advisory Committee) has not supported population-based screening for prostate cancer [7].

In the vast majority of practices Māori men were less likely to be screened, however, two Māori provider practices showed relatively equal screening rates for Māori and non-Māori men. Concurrently, Māori provider practices showed the lowest overall screening rates among the 31 practices. Māori provider practices differed from other provider practices with respect to a higher proportion of Māori patients but they were also smaller in terms of both number of patients and GPs. With the knowledge that screening is mainly GP-driven, it is unclear why GPs are less likely to initiate prostate cancer screening for Māori men. More research is needed into reasons for the 50% lower screening rates for Māori versus non-Māori men in New Zealand.

### Strengths and limitations

The strength of this study is that population-based data were acquired from the general practice computerised records, which allowed us to distinguish the reasons for PSA testing, and thus calculate rates for screening compared with testing due to symptoms or disease monitoring. In addition, we were able to link the practice-based records through unique NHI numbers to laboratory data and prostate cancer registrations, meaning that we could cross-validate information from practices and acquire comprehensive history on PSA testing and follow-up care, including specialist referrals and biopsy results. One of the main advantages of this study was sufficient numbers of Māori men for statistical evaluation of the results.

We identified four potential limitations to our study: Firstly, practice notes might not be complete, particularly regarding reasons for PSA testing. Secondly, we did not record information on symptoms for men without an elevated PSA; we estimated the proportion of screened men based on information about elevated PSA tests or biopsies prior to 2010. Therefore, the overall number of screened men may be overestimated. In a pilot study based on data from five general practices in the Midland Cancer Network region examining reasons for testing in all men with PSA test in 2010, 11% of men were tested due to symptoms, and the proportion of screened men was 75% compared with 85% in our study [13].

Thirdly, we have considered men enrolled in the general practices in 2010 as the baseline population, not men who visited their GP in that year. However, a current NZ Health Survey showed that more than 80% of men older than 40 years visited their GP in 2011/2012 and Māori men were as likely to visit their GPs as non-Māori men [41].

Fourthly, the sample size was probably not sufficient for robust analysis of secondary outcomes, i.e. referral and biopsy rates. However, we believe that the presented findings will guide further research into the outcomes of screening for prostate cancer in New Zealand.

## Conclusions

This study makes an important contribution to New Zealand’s national dialogue on prostate cancer inequities between Māori and non-Māori men, prostate cancer screening and cancer inequities in general. We found that lower PSA screening rates in Māori men are a major determinant of a lower prostate cancer incidence. Due to potential harms of prostate cancer screening [7], it is not clear whether Māori men would benefit from increased screening rates. Māori men were more likely to have an elevated PSA result compared with non-Māori men, suggesting that a larger proportion of Māori men was at risk of prostate cancer compared with non-Māori men. There was a large variation in PSA screening rates among practices, with Māori provider practices showing the lowest overall screening rates. Although there were no differences between Māori and non-Māori men in the proportion of follow-up investigations, overall less than half of the men with an elevated PSA result were referred to a specialist for further examination. The large variation in screening patterns among practices may be partly attributed to inconsistent recommendations for screening by various organisations, but this argument would not hold for the observed differences by ethnicity. One recommendation that has been found to be consistent for a majority of organisations is that decisions around screening should be made following an in-depth discussion about the pros and cons of screening between the patient and his health care provider. Screening in New Zealand is currently mainly GP-driven, so there is obviously a need for more education for GPs, probably more discussion time between GPs and patients around screening and prostate cancer detection, and for strategies leading to improvements in health literacy, thereby creating an environment, in which shared decision-making between GPs and patients can be achieved.

More research is needed to better understand GP decision-making about screening pathways for prostate cancer, which will in turn help to further clarify the causes of higher mortality rates for Māori compared with non-Māori men with prostate cancer. To sum up, opportunistic screening is relatively common in New Zealand but varies widely by practice and ethnicity. Better understanding of prostate cancer risk on an individual basis, employing shared decision-making regarding screening and consistent follow-up strategies for men considered to be at higher risk of prostate cancer may help to target resources and thus improve outcomes, particularly for Māori men in the future. The ultimate goal should be to devise strategies for prostate cancer control that unite best practice, equity, and cost-effectiveness of health care services.
